# The Cross-Sectional Association of Energy Intake and Dietary Energy Density with Body Composition of Children in Southwest China

**DOI:** 10.3390/nu7075228

**Published:** 2015-07-03

**Authors:** Xue Zhou, Hongmei Xue, Ruonan Duan, Yan Liu, Lishi Zhang, Louise Harvey, Guo Cheng

**Affiliations:** 1 Department of Clinical Nutrition, Sichuan Provincial Academy of Medical Sciences & Sichuan Provincial People’s Hospital, Chengdu 610072, Sichuan, China; E-Mail: zx_sophia@163.com; 2 Department of Nutrition, Food Safety and Toxicology, West China School of Public Health, Sichuan University, Chengdu 610041, Sichuan, China; E-Mails: xhmei_1109@163.com (H.X.); Duanrn121@163.com (R.D.); Liuyan_liya@163.com (Y.L.); lishizhang_56@163.com (L.Z.); 3 Danone Nutricia Early Life Nutrition, Singapore 138671, Singapore; E-Mail: Louise.HARVEY@danone.com

**Keywords:** energy intake, dietary energy density, body composition, obesity, children

## Abstract

Objective: We examined whether dietary energy intake (EI) and dietary energy density (ED) were cross-sectionally associated with body composition of children living in Southwest China. Design and Methods: Multivariate regression analyses were performed on three day, 24 h dietary recall data and information on potential confounders from 1207 participants aged 8–14 years. EI was calculated from all foods and drinks and ED was classified into five categories. Body mass index (BMI) z-scores, percentage of body fat (%BF), fat mass index (FMI), fat-free mass index (FFMI) and ratio of waist to hip circumference (WHR) were used to describe body composition. Results: Boys with higher total EI had higher BMI z-scores, %BF, and FMI than boys with lower total EI both before and after measurements were adjusted for confounders (age, fiber intake, physical activity, the timing of adding complementary foods, paternal education level and maternal BMI) (*p* ≤ 0.04). However, EI was not associated with body composition in girls. Dietary ED, in any category, was not associated with body composition in either gender. Conclusions: Dietary ED was not associated with body composition of children in Southwest China, while dietary EI in boys, not girls, was positively associated with body composition. Reducing dietary energy intake may help to prevent obesity and related diseases in later life among boys living in Southwest China.

## 1. Introduction

The epidemic of childhood adiposity has been reported in both developed [1] and developing countries [2]. Childhood overweight/obesity is widely recognized as a risk factor for diabetes [3], coronary heart disease [4], hypertension [5], dyslipidemia [6], and metabolic syndrome [7] in childhood or later life.

Obesity is known to occur as a result of genetic, environmental, life style and behavioral influences [8]. Dietary energy intake (EI) is a major environmental risk factor which has been the focus of numerous studies of European [9], American [10], Australian [11], Canadian [12], Italian [13] and Chinese children [14]. However, described associations of dietary EI with child body composition have been inconsistent in these studies. With the increased consumption of high energy dense foods over the past decades [15], the impact of high energy dense food, or daily dietary energy density (ED), on childhood obesity has received increasing attention. In adulthood, high ED diets may challenge appetite control systems [16] and lead to overeating and becoming overweight [17,18]. The World Health Organization (WHO) recommends restricting high energy dense foods (e.g., chocolate, sugar, butter and snacks) as a way to prevent the obesity epidemic [19] and the American Academy of Pediatrics also encourages children to consume low energy dense foods (e.g., fruits and vegetables) and recommends a macronutrient-balanced diet [20,21]. Epidemiological studies in children from the United States (U.S.) found that higher ED diets were associated with higher EI [22,23]. Moreover, dietary ED was commonly interpreted as a marker of diet quality. Interestingly, children in Germany with lower diet quality were found to enter puberty earlier which was considered as a risk factor for obesity [24]. Yet, the influence of ED on body composition is inconsistent in existing epidemiological studies. ED was found to be associated with overweight or body fat mass in two cross-sectional studies of U.S. [23] and Japanese children [25], and in multiple prospective studies of British children [26,27,28], however, there was no relationship between ED and body mass index (BMI), weight status, or excess adiposity in these studies among European [9], Danish [29], German [30] and U.S. [31,32,33] children. In Irish children the results regarding the association between ED and body composition were different because the methods to calculate ED and define obesity are different [34]. These inconsistent results may reflect the different lifestyles of the populations examined, misreporting of energy intake [35] and the methodological challenges inherent in accurately assessing EI and calculating dietary ED [36].

In China, the prevalence of childhood obesity has increased dramatically over the last 20 years [2]. The dietary pattern of Chinese children has also changed during this period, a change which can be characterized by increased consumption of energy-dense, micronutrient-poor foods *i.e.*, snacks and processed food [37]. It is possible that the overconsumption of high energy dense foods or high ED diet may contribute to the epidemic of child adiposity in China. Identifying the potential relevance of dietary EI and ED on body composition of Chinese children may help prevent obesity and related diseases in later life.

Thus, using data collected from children living in Sichuan, Southwest China, we aimed to investigate whether dietary EI or ED was associated with body composition by assessing BMI z-scores, percentage of body fat (%BF), fat mass index (FMI), fat-free mass index (FFMI) and ratio of waist to hip circumference (WHR). In order to overcome the limitations of previous studies, the present study took many precautions to guarantee the accuracy of the results. These precautions included eliminating the misreporting of energy intake, assessing EI for not only all foods but also all drinks (including caloric beverages), calculating ED using five approaches, controlling for physical activity level, and analyzing a range of various potential confounders.

## 2. Subjects and Methods

### 2.1. Study Sample

Participants for this analysis were from the cross-sectional study “Diet Quality During Childhood”, which aimed to obtain information on the diet, anthropometry and life style (e.g., physical activity) of children in Southwest China. The survey aimed to recruit children representative of Southwest China. To this end, two elementary schools and two middle schools were chosen from 35 elementary schools and 47 middle schools using cluster random sampling. Schools were located in Chengdu in Sichuan, a large, representative city in Southwest China. Potential subjects were chosen from different classes in the above four schools using stratified sampling. Inclusion criteria for subjects to be invited to participate included: Singleton birth; birth weight > 2500 g; healthy population (not undergoing medical treatment or therapy related to management of body weight); and living with parents in the local community. We did not include multiple births or infants with a low birth weight as we were concerned that children from these situations may differ in their health and development, potentially displaying deviating growth patterns, and may have undergone special feeding regimes. Parents and children were informed and the parent of each child provided written informed consent. The study protocol was approved by the Ethics Committee of the Sichuan University.

### 2.2. Dietary Data

Dietary data were collected in schools, using three day, 24 h dietary recalls administered by well-trained investigators, which asked participants aged 10 years or older to recall all food (including foods eaten outside the home and nutrition supplements) and beverage consumption over two weekdays and one weekend day. In the interviews, children were asked to recall all foods and beverages consumed as well as the corresponding timing. Children younger than 10 years were asked to describe their own food intake with assistance from investigators and the person’s responsible for preparing the child’s meals on weekdays. For the weekend day food record for these children, parents were instructed to complete a food record by directly pre-weighing all foods and beverages that were served to their children, and weighing the leftovers. A short questionnaire was included to help clarify more difficult foods, such as type of oil used. Special attention was given to the estimation of serving sizes in terms of household measures. During the interviews, food models for foods commonly consumed by children were provided. In addition, a photobook containing names and photos of snacks, as well as a picture of the serving size in the common commercial packet (e.g., carton) was provided in the interviews as a reference. All dietary information was managed using a validated nutrient analysis software (NCCW, version 2014), developed by Qingdao University. Mean daily dietary energy and nutrient intakes from all foods and drinks were calculated using NCCW based on the China food composition database [38].

### 2.3. Misreporting of EI and Calculating of Dietary ED

The information on physical activity was also carefully collected in schools by well-trained investigators using a questionnaire. Physical activity was converted to daily energy expenditure by metabolic equivalent (MET) [39] and grouped into tertiles (T1–T3) as low/moderate/high activity levels. Individualized estimated energy requirements (EER) were calculated using sex-specific equations based on body weight and age-related coefficients for the energy requirements of growth (adding 8.6 kJ (2 kcal) for each gram of weight gained during growth) [40]. Activity levels for each child were used to adjust the EER by ±15% in children with high or low physical activity levels compared to moderate levels [40]. To take into account the variation inherent in the methods used to estimate EI and EER, a 95% CI for the accuracy of EI/EER was calculated [41]; this 95% CI was 0.78–1.22. Since energy under-reporting may affect the real relationship between dietary EI/ED and body composition [42], any children with an EI/EER < 0.78 were defined as under-reporters (6.8% of the total sample) and excluded from the analysis. Therefore, except for under-reports, other EI reports are plausible energy reports in this analysis.

The daily dietary ED (kJ/g) was computed by dividing total food energy by total food weight. Different ED categories may affect the relationship between the variables and outcomes [36] but, at present, there is no standardized method for which types of energy sources (e.g., solid foods, water, beverages, or all of them) should be used to calculate ED. Thus, based on previous studies [36,43], we applied a total of five calculations in our analysis. ED_1_ contained all solid foods and drinks. ED_2_ contained solid foods only excluded all drinks, including all energy-containing drinks or energy-free drinks, which accounted for much of the variability in energy density by providing a significant amount of weight with no or little energy [44]. ED_3_ included all solid foods and milk, milk as a drink, but no other beverages. It was necessary to include milk in the calculation of ED because lots of foods, such as breakfast cereals, are typically eaten with milk [45]. ED_4_ included solid foods and energy-containing beverages (>21 kJ or 5 kcal per 100 g) [46], e.g., soft drinks, which have been studied as a risk factor for higher rates of overweight and obesity [47] and thus should be taken into account in our analysis. Finally, ED_5_ included solid foods, and milk and energy-containing beverages but no other beverages.

### 2.4. Anthropometry

Anthropometric measurements of the participants were performed by well-trained investigators according to standard procedures [48] in a private room of the school. Children were dressed in underwear only and were barefoot. Standing height was measured in duplicate to the nearest 0.1 cm and weight was measured in duplicate to the nearest 0.1 kg by using an Ultrasonic Weight and Height Instrument (DHM-30, Dingheng Ltd., Zhengzhou, China). In addition, waist circumference at the umbilicus and hip circumference at the most prominent parts of the hip were measured in duplicate to the nearest 0.1 cm by a tape measure. Skinfold thickness was measured on the right side of the body at triceps and subscapular angle sites to the nearest 0.1 mm using a Holtain caliper (Holtain Ltd., Crymych, UK).

In this analysis, BMI was calculated as weight/height^2^ (kg/m^2^). Sex- and age-specific BMI z-scores were calculated using the equation from Cole *et al.* [49] based on the centile curves for Chinese children and adolescents [50]. Overweight/obesity was defined according to both the Working Group on Obesity in China (WGOC) [51] and the International Obesity Task Force (IOTF) BMI cut-offs for children [49]. The %BF was calculated using the Slaughter equations [52]. Since %BF has recently been criticized as inadequately reflecting body-size-adjusted adiposity [53], FMI ((weight × %BF)/height^2^) and FFMI ((weight − (weight × %BF))/height^2^), as introduced by Maynard *et al.* [54], were calculated as measures of body composition. The ratio of waist-to-hip (WHR) was also estimated [55].

### 2.5. Other Data

Family related information (e.g., parental education level), child birth weight, exclusive breastfeeding duration as well as the timing of adding complementary foods and parental weight and height were self-reported via questionnaires sent to the parents one week prior to the interviews.

### 2.6. Statistical Analysis

All statistical analyses were carried out using Statistical Analyses System (SAS, versions 9.1). A *p*-value < 0.05 was considered statistically significant, except for the analysis of interaction, where *p* < 0.1 was considered significant. Normality of the continuous variables was assessed by statistical testing and graphical inspection. Dietary ED and EI were the primary exposures of interest. As there was an interaction between sex and the relationship between dietary ED/EI and body composition in the participants, we analyzed data from boys and girls separately.

Differences in anthropometric, dietary and family data, between boys and girls, were tested using *t* tests for normally distributed continuous variables, Wilcoxon rank sum tests for non-normally distributed continuous variables and chi-square tests for categorical variables. To investigate the potential association between dietary EI/ED and body composition, multivariate regression models were used. We calculated adjusted mean outcome levels (BMI z-scores, %BF, FMI, FFMI and WHR) in tertiles of dietary EI/ED (T1–T3). Potential confounders that were considered include: age, birth weight; exclusive breastfeeding duration (≥4 months, yes/no); the timing of adding complementary foods (≥4 months, yes/no); physical activity (low/moderate/high); parental education level (≥12 years school education, yes/no); overweight parental BMI (BMI ≥ 24 kg/m^2^, yes/no); smoking in the house (yes/no); the percentage of EI from protein, fat, carbohydrate, and fiber intake. Each variable was initially considered separately; variables which showed an independent significant effect in the basic models or substantially modified the association between EI/ED and body composition markers were included in the multivariate analyses. The adjusted means were the least-squares means predicted by the model when the other variables were held at their mean values. Tests by trend were performed using mean daily dietary EI/ED intakes as continuous variables.

## 3. Results

A total of 2170 healthy children (boys = 1118, girls = 1052) aged 8–14 years living in Sichuan were recruited. Among them, 87 children declined to participate in the study, giving a response rate of 96%. Energy reporting, presence of complete personal data (age, birth weight, exclusive breastfeeding duration, the timing of adding complementary foods and physical activity) and family information were considered in the analysis. Thus, 206 children (boys = 106, girls = 100) were excluded for implausible energy reporting and 670 children (boys = 400, girls = 270) were also excluded for missing family information or personal data. In total, 1207 (boys 572, girls 635) children, who had plausible energy reporting as well as complete family information and personal data, were included in our analysis.

Overall, girls had higher BMI z-scores, %BF and FMI than boys, while boys had higher FFMI and WHR than girls (*p* ≤ 0.02, Table 1). Furthermore, boys were more overweight/obese than girls according to both the WGOC (*p* = 0.0004) and IOTF (*p* = 0.002) definitions of overweight/obesity. ED ranged from 4.0 to 6.9 (kJ/g·d) and depended on category. Significant differences in intake of energy, fiber and ED from each category were observed between boys and girls (*p* ≤ 0.003).

**Table 1:** Early life, anthropometric, dietary and family characteristics of children aged 8–14 years ^1^.

	Boys	Girls	*p* ^2^
*n* (%)	572 (47%)	635 (53%)	
Age (year)	12.3 ± 0.2	11.7 ± 0.9	0.4
Birth weight (kg)	3.3 ± 0.5	3.2 ± 1.0	<0.0001
Exclusive breastfeeding duration ( *n*, %) ^3^	435 (76.1)	483 (76.1)	0.9
The timing of adding complementary foods (*n*, %) ^3^	475 (83.0)	518 (81.6)	0.5
High physical activity level (*n*, %)	177 (30.9)	190 (29.9)	0.7
**Anthropometric Data ^4^**			
BMI *z*-scores	0.1 (−0.4, 0.8)	0.3 (−0.2, 0.9)	0.0003
%BF	14.5 (11.5, 19.8)	18.4 (14.8, 24.2)	<0.0001
FMI (kg/m^2^)	2.8 (2.1, 4.1)	3.1 (2.3, 4.1)	0.02
FFMI (kg/m^2^)	14.1 (13.0, 15.8)	13.8 (12.5, 15.7)	0.006
WHR	0.85 (0.82, 0.89)	0.83 (0.79, 0.86)	<0.0001
Overweight/obesity ( *n*, %) ^5^	118 (20.6)	83 (13.1)	0.0004
Overweight/obesity (*n*, %) ^6^	98 (17.1)	69 (10.9)	0.002
**Dietary Data ^7^**			
Total energy intake (MJ/day)	8.4 (6.9, 10.5)	7.6 (6.3, 9.4)	<0.0001
Carbohydrate (% of energy)	58.7 (52.8, 64.5)	59.8 (53.6, 64.4)	0.8
Fat (% of energy)	26.8 (21.8, 32.0)	25.8 (21.3, 31.6)	0.7
Protein (% of energy)	14.5 (12.7, 16.5)	14.4 (12.9, 16.4)	0.06
Fiber intake (g/MJ)	1.0 (0.7, 1.2)	1.1 (0.8, 1.4)	<0.0001
ED_1_ (kJ/g·day)	4.2 (3.7, 5.0)	4.0 (3.5, 4.6)	<0.0001
ED_2_ (kJ/g·day)	6.9 (6.0, 7.9)	6.6 (5.9, 7.6)	0.002
ED_3_ (kJ/g·day)	5.9 (5.2, 6.8)	5.7 (5.0, 6.5)	0.0003
ED_4_ (kJ/g·day)	6.6 (5.9, 7.7)	6.4 (5.7, 7.3)	0.003
ED_5_ (kJ/g·day)	5.7 (5.1, 6.5)	5.5 (4.9, 6.3)	0.0006
**Family Data**			
Paternal education level (*n*, %) ^8^	137 (24.0)	160 (25.2)	0.6
Maternal education level ( *n*, %) ^8^	100 (17.5)	132 (20.8)	0.2
Paternal BMI (kg/m^2^)	23.1 ± 2.7	23.4 ± 2.9	0.8
Paternal overweight ( *n*, %) ^9^	221 (38.6)	246 (38.7)	0.9
Maternal BMI (kg/m^2^)	21.8 ± 2.7	21.9 ± 3.0	0.8
Maternal overweight ( *n*, %) ^9^	112 (19.6)	132 (20.8)	0.6
Smoking in the house ( *n*, %) ^10^	384 (67.1)	420 (66.1)	0.7

^1^ Values are means ± SD, medians (Q1, Q3) or frequency. ^2^ Tests for difference between boys and girls were performed using *t* tests for normally distributed continuous variables, Wilcoxon rank sum tests for non-normally distributed continuous variables, and chi-square tests for categorical variables. ^3^ *n*, the number of participants who were exclusively breastfeed for ≥4 months or had addition of complementary foods from ≥4 months of age; %, in terms of percentage. ^4^ BMI (body mass index) z-scores was calculated using the equation according to Cole *et al.* [49]; %BF, percentage body fat calculated according to Slaughter equations [52]; FMI, fat mass index ((weight × %BF)/height^2^); FFMI, fat-free mass index ((weight – (weight × %BF))/height^2^); WHR, ratio of waist-to-hip circumference. ^5^ Overweight/obesity defined according to the Working Group on Obesity in China (WGOC) [56]. ^6^ Overweight/obesity defined according to the International Obesity Task Force (IOTF) [49]. ^7^ ED_1_ (dietary energy density), all solid foods and drinks; ED_2_, solid foods only; ED_3_, solid foods and milk, milk as a drink, but no other beverages; ED_4_, solid foods and energy-containing beverages (>21kJ/100g) [46]; ED_5_, solid foods and milk and energy-containing beverages. ^8^ *n*, the number of participants whose parents had school education ≥12 years; %, in terms of percentage. ^9^ BMI (in kg/m^2^) ≥ 24 [57].^10^ n, the number of participants whose house contained a person who smokes; %, in terms of percentage.

Boys with higher total energy intake had higher BMI z-scores, %BF, FMI and FFMI than boys with lower total energy intake (*p* ≤ 0.03, Table 2). These associations were maintained (*p* ≤ 0.04) after the addition of confounders to the model (adjusted model), which indicating that, except for FFMI (*p* = 0.4), age, fiber intake, physical activity level, the timing of adding complementary foods, paternal education level and maternal BMI did not explain the relationship between EI/ED and weight status. By contrast, energy intake was not associated with body composition in girls in both the unadjusted and adjusted model.

**Table 2:** Association of total energy intake with BMI *z*-scores, %BF, FMI, FFMI and WHR among children aged 8–14 years ^1^.

	Total Energy Intake ^2^			*p* for
	T1	T2	T3	Trend ^3^
Boys *n* = 572	190	191	191	
BMI *z*-scores				
Unadjusted	0.1 (−0.0, 0.3)	0.3 (0.1, 0.4)	0.4 (0.3, 0.5)	0.02
Adjusted ^4^	−0.0 (−0.2, 0.2)	0.2 (−0.0, 0.4)	0.3 (0.1, 0.6)	0.003
%BF				
Unadjusted	15.6 (14.4, 16.8)	17.8 (16.6, 19.0)	18.1 (16.9, 19.3)	0.01
Adjusted ^5^	16.2 (14.3, 18.1)	17.6 (15.6, 19.6)	17.9 (15.9, 19.9)	0.04
FMI				
Unadjusted	3.1 (2.8, 3.3)	3.3 (3.0, 3.5)	3.6 (3.4, 3.8)	0.001
Adjusted ^5^	3.3 (2.9, 3.6)	3.3 (3.0, 3.7)	3.7 (3.3, 4.0)	0.01
FFMI				
Unadjusted	14.1 (13.8, 14.5)	14.4 (14.1, 14.8)	14.7 (14.4, 15.1)	0.03
Adjusted ^5^	14.0 (13.5, 14.6)	14.4 (13.8, 14.9)	14.4 (13.8, 14.9)	0.4
WHR				
Unadjusted	0.85 (0.84, 0.86)	0.86 (0.85, 0.87)	0.86 (0.85, 0.86)	0.7
Adjusted^4^	0.84 (0.83, 0.85)	0.85 (0.84, 0.87)	0.85 (0.84, 0.86)	0.3
Girls *n* = 635	211	212	212	
BMI *z*-scores				
Unadjusted	0.3 (0.2, 0.4)	0.4 (0.3, 0.5)	0.5 (0.4, 0.6)	0.2
Adjusted ^4^	0.3 (0.1, 0.5)	0.4 (0.1, 0.6)	0.5 (0.2, 0.7)	0.3
%BF				
Unadjusted	19.2 (18.3, 20.1)	20.2 (19.3, 21.1)	20.5 (19.6, 21.4)	0.4
Adjusted ^5^	19.6 (18.3, 21.0)	20.0 (18.7, 21.4)	20.1 (18.7, 21.5)	0.8
FMI				
Unadjusted	3.4 (3.2, 3.6)	3.5 (3.3, 3.7)	3.3 (3.1, 3.5)	0.6
Adjusted ^5^	3.3 (3.0, 3.6)	3.3 (3.0, 3.6)	3.1 (2.7, 3.4)	0.2
FFMI				
Unadjusted	13.8 (13.5, 14.1)	14.1 (13.8, 14.4)	14.5 (14.2, 14.9)	0.1
Adjusted ^5^	14.0 (13.5, 14.5)	14.1 (13.6, 14.6)	14.5 (14.0, 15.0)	0.3
WHR				
Unadjusted	0.82 (0.82, 0.83)	0.83 (0.82, 0.84)	0.83 (0.82, 0.84)	0.2
Adjusted ^4^	0.82 (0.81, 0.83)	0.83 (0.82, 0.84)	0.83 (0.82, 0.84)	0.2

^1^ Values are model-adjusted least-squares means; 95% CIs in parentheses. Tertiles (T) are based on mean daily energy intakes by three day, 24 h dietary recalls. Body mass index z-scores, BMI z-scores; percentage body fat, %BF; fat mass index, FMI; fat-free mass index, FFMI; ratio of waist-to-hip circumference, WHR. ^2^ Ranges (minimum − maximum) for tertiles 1 through 3, respectively—boys: 3.56–7.35, 7.38–9.71, and 9.72–21.85 MJ/day; girls: 3.36–6.72, 6.74–8.57, and 8.61–22.66 MJ/day. ^3^ Linear trends (p for trend) were tested with mean daily energy intake as continuous variables. ^4^ Adjusted for age, fiber intake, the timing of adding complementary foods, paternal education level and maternal BMI. ^5^ Adjusted for age, fiber intake, physical activity, paternal education level and maternal BMI.

ED, in any category, was not significantly associated with any outcomes in either boys or girls. Thus, we show only one table describing the association between ED of all solid foods and drinks (ED_1_) and body composition (Table 3).

**Table 3:** Association of ED of all solid foods and drinks with BMI *z*-scores, %BF, FMI, FFMI and WHR among children aged 8–14 years ^1^.

	ED of All Solid Foods and Drinks ^2^			*p* for
	T1	T2	T3	Trend ^3^
Boys *n* = 572	190	191	191	
BMI *z*-scores				
Unadjusted	0.3 (0.1, 0.4)	0.3 (0.1, 0.4)	0.2 (0.1, 0.4)	0.9
Adjusted ^4^	0.2 (−0.1, 0.4)	0.2 (−0.1, 0.4)	0.1 (−0.1, 0.3)	0.9
%BF				
Unadjusted	17.4 (16.2, 18.6)	17.4 (16.2, 18.6)	16.6 (15.4, 17.9)	0.7
Adjusted ^5^	17.2 (15.2, 19.2)	17.3 (15.4, 19.2)	16.7 (14.8, 18.7)	0.9
FMI				
Unadjusted	3.2 (3.0, 3.4)	3.4 (3.1, 3.6)	3.4 (3.1, 3.6)	0.2
Adjusted ^5^	3.3 (2.9, 3.7)	3.4 (3.1, 3.8)	3.5 (3.1, 3.8)	0.2
FFMI				
Unadjusted	14.7 (14.3, 15.0)	14.5 (14.2, 14.9)	14.3 (14.0, 14.7)	0.4
Adjusted ^5^	14.4 (13.8, 14.9)	14.3 (13.7, 14.8)	14.1 (13.5, 14.6)	0.4
WHR				
Unadjusted	0.86 (0.85, 0.86)	0.85 (0.85, 0.86)	0.85 (0.85, 0.86)	0.4
Adjusted ^4^	0.85 (0.83, 0.86)	0.85 (0.83, 0.86)	0.84 (0.83, 0.86)	0.4
Girls *n* = 635	211	212	212	
BMI z-scores				
Unadjusted	0.3 (0.2, 0.5)	0.4 (0.3, 0.5)	0.5 (0.4, 0.6)	0.5
Adjusted ^4^	0.3 (0.1, 0.5)	0.4 (0.1, 0.6)	0.5 (0.3, 0.7)	0.3
%BF				
Unadjusted	19.6 (18.7, 20.6)	19.8 (18.9, 20.7)	20.5 (19.6, 21.4)	0.5
Adjusted ^5^	19.5 (18.2, 20.8)	19.7 (18.4, 21.1)	20.6 (19.2, 22.0)	0.4
FMI				
Unadjusted	3.4 (3.2, 3.6)	3.4 (3.2, 3.6)	3.5 (3.3, 3.7)	0.9
Adjusted ^5^	3.2 (2.9, 3.5)	3.2 (2.9, 3.5)	3.3 (3.0, 3.6)	0.9
FFMI				
Unadjusted	14.0 (13.6, 14.3)	14.1 (13.8, 14.4)	14.3 (14.0, 14.6)	0.3
Adjusted ^5^	14.0 (13.5, 14.5)	14.2 (13.7, 14.7)	14.4 (13.9, 14.9)	0.2
WHR				
Unadjusted	0.83 (0.83, 0.84)	0.82 (0.82, 0.83)	0.83 (0.82, 0.83)	0.06
Adjusted ^4^	0.83 (0.82, 0.84)	0.82 (0.81, 0.84)	0.83 (0.82, 0.84)	0.2

^1^ Values are model-adjusted least-squares means; 95% CIs in parentheses. Tertiles (T) are based on mean daily dietary energy density (ED) of all solid foods and drinks by three day, 24 h dietary recalls. Body mass index *z*-scores, BMI *z*-scores; percentage body fat, %BF; fat mass index, FMI; fat-free mass index, FFMI; ratio of waist-to-hip circumference, WHR. ^2^ Ranges (minimum–maximum) for tertiles 1 through 3, respectively—boys: 1.63–3.87, 3.87–4.67, and 4.67–7.70 (kJ/g·day); girls: 1.64–3.70, 3.70–4.36, and 4.36–9.47 (kJ/g·day). ^3^ Linear trends (*p* for trend) were tested with mean daily dietary ED of all solid foods and drinks as continuous variables. ^4^ Adjusted for age, fiber intake, the timing of adding complementary foods, paternal education level and maternal BMI. ^5^ Adjusted for age, fiber intake, physical activity, paternal education level and maternal BMI.

In addition, children with missing data were analyzed. No significant differences were observed between the participants who had completed information and those who were missing family or personal data, in terms of age, BMI z-scores, FMI, FFMI, %BF and WHR and nutrient intakes (data not shown).

## 4. Discussion

Our study shows that EI was associated with body composition of boys, but that this association was not evident in girls. Our study does not support a relationship between dietary ED and body composition in children.

Recently, a cross-sectional study among Chinese children aged 3–6 years showed that EI of main meals was associated with being overweight [14], a result which supports our finding. Furthermore, cross-sectional studies from other countries have suggested that EI is positively associated with BMI *z*-scores in 2–9 year old European children [9], whilst not associated with BMI or waist circumference in children aged 5–17 years in Australia [11]. In prospective studies, higher daily EI at 4 years of age was found to be significantly related to being overweight at 6 years of age in Canadian children [12]; however, the association was not found in 8 year old Italian children in a 4 year follow up [13]. We speculate that these inconsistent findings are likely due to different ethnicities, economic status, and social environments between countries. In the present analysis, EI was found to be positively associated with body composition of boys, which is consistent with a cross-sectional study that found that dietary EI had an effect on BMI percentile only in 6–11 year old American boys [10]. Our findings may stem from the existing significant difference in body composition between the two genders in the children included in our study. In our cohort, boys were more overweight/obese than girls. In addition, we found that boys consumed more energy and had higher dietary ED than girls, which may be due to special dietary behavior in girls, who may have been attempting to decrease their weight. In our view, such phenomena is more common in females [58] and might explain, at least partly, why the expected association between EI and body composition was observed only in boys but not in girls. Furthermore, another important factor is that our study included both pre-pubertal and pubertal children. Puberty influences body composition and might be more important in girls than in boys across this age range. The absence of adjustment for this variable could explain, at least in part, why the association between EI and body composition was less clear in girls.

In the present analysis, ED was not significantly associated with the body composition of the children. Our finding is similar to a cross-sectional study which reported that ED was not associated with BMI *z*-scores of 2–9 year old European children [9]. In contrast, dietary ED was found to be cross-sectionally associated with being overweight in boys aged 6–15 years in Japan [25] and with body weight status in 2–8 year old children [23] and risk factors for adiposity in children [22] in America. A prospective association between dietary ED at age 7 years and FMI at age 9 years was found in British children [28]; in a larger sample of the same population, each increase of 1 kJ/g dietary ED at 10 years was associated with 0.16 ± 0.06 kg more fat mass at age 13 years [26]. However, other previous prospective observational studies suggested that dietary ED was not associated with: Weight status among U.S children [31,32,33]; three year weight gain in Danish children [29]; or BMI and FMI *z*-scores in Germen children [30]. In order to overcome the limitations of prior studies, such as difficult to assess EI or lack of standardized methods for calculating dietary ED, our study took precautions to ensure the accuracy of dietary data including assessing EI for all foods and all drinks (including caloric beverages) and calculating ED using five approaches. However, despite these precautions, our study still could not find an association between dietary ED, in any category, and body composition in children.

Interestingly, a study which used different methods of calculating ED found that only dietary ED which excluded all or most beverages was positively associated with changes in FMI from age 6–8 years to 13–17 years in Irish children, while ED calculated by any method was not associated with %BF, BMI or waist circumference z-scores in the same sample [34]. Given the recommendations from the WHO to restrict intake energy dense foods as a measure to aid in preventing an obesity epidemic [19], our findings seem surprising. However, if we note that the evidence for the WHO recommendations was based primarily on treatment intervention studies [59,60] e.g., decreasing the ED of a meal significantly decreases meal energy intake and body weight among obese children, our findings can be accepted. On the other hand, our findings may stem from the different economic status and social environment of China compared to other countries. In contrast to developed countries [22], low energy-dense food, such as vegetables and fruits, are freely available and affordable for low income families in China. Although increased intake of high energy dense foods (e.g., snacks) in the daily diet have been reported over the past few decades in China, this trend is mainly confined to urban, higher income, and educated populations [37]. In our study, all of the participants were from Sichuan, which is in Southwest China, where economic development has been slower than coastal cities in Northeast China. Dietary patterns may not change as fast as in the developed cities and consumption of high energy dense foods may not have increased as much in our participants. Thus, our findings suggest that children living in southwest China may not gain excess body weight regardless of the energy density of their diet.

There are some limitations to our study. First, the data from this study is cross-sectional. Therefore, although a strong association was found between EI and body composition in boys, we were not able to determine a cause-effect relationship between dietary EI and child weight gain. However, this is the first study which focused on the relationship between dietary ED and body composition in children living in China and we plan to conduct a follow up study to identify potential mechanisms. Second, the 24 h dietary recall method applied in our study has some limitations as it is relies on the participants’ memories, particularly for the children less than 10 years of age. However, 24 h dietary recalls are considered appropriate for studying energy and nutrient intake in large samples of children and are less burdensome and invasive than food records [61]. For the children aged less than 10 years, we tested a series of measures during our preliminary experiments to make sure that the collected dietary data from this special age group was reliable and suitable for analysis. The validity of the method was confirmed by preliminary experiments in our study, in which we compared the data collected by dietary recall with the three day weighed food records and found no statistically significant difference between the results. To avoid arbitrary recalled diet data, dietary recalls were conducted on two weekdays and one weekend day in our study. Finally, the sample used in our analysis is not as large as that used in other cross-sectional studies. However, our analysis did not contain any energy under-reporters and was carefully controlled with respect to potential confounders. In addition, even though the sample was not as large, it was almost sufficient to identify the relationship between dietary EI and body composition in boys by statistical power of 0.67.

Our study has several strengths. First, physical activity was taken into account as a confounder in the analysis. Second, some energy-dense foods e.g., snacks, are more likely to be “missed out” among overweight and obese children in a diet survey [62] which leads to under-reporting of EI/ED estimates [42]. Thus, in our analysis, EI under-reporters were excluded and physical activity was used to adjust the EER for different activity levels, in order to guarantee the accuracy of the energy reporter categories. The current inconsistences about the relations, or lack of the relations, of the dietary ED and body composition in children may be due to the methods used to calculate dietary ED and the assessment/classification of being overweight or obese [34,36]. In our analysis, dietary data was recorded in detail (including caloric/non-caloric beverage and water intake). EI was carefully estimated for all foods and drinks, and ED was calculated by five approaches. The anthropometric data was based on duplicate measurements instead of reported data and five body composition markers were calculated. In addition, food models for foods commonly consumed by children and a photobook (containing approximately 200 food items) were provided to help ensure the accuracy of assessment of portion size in the daily diet. Finally, we analyzed a range of various potential confounders in our study.

## 5. Conclusions

Our data identified that EI was positively associated with body composition in boys, while dietary ED was not associated with body composition in either boys or girls. Reducing dietary energy intake may help to prevent obesity and related diseases in later life among boys living in Southwest China.
